# Transcobalamin II Deficiency in Four Cases with Novel Mutations

**DOI:** 10.4274/tjh.2014.0154

**Published:** 2015-12-03

**Authors:** Şule Ünal, Tony Rupar, Sevgi Yetgin, Neşe Yaralı, Ali Dursun, Türkiz Gürsel, Mualla Çetin

**Affiliations:** 1 Hacettepe University Faculty of Medicine, Division of Pediatric Hematology, Ankara, Turkey; 2 Victoria Hospital, London Health Sciences Centre, Biochemical Genetics Laboratory, London, Canada; 3 Ankara Children’s Hematology and Oncology Hospital, Clinic of Pediatric Hematology, Ankara, Turkey; 4 Hacettepe University Faculty of Medicine, Division of Metabolism and Nutrition, Ankara, Turkey; 5 Gazi University Faculty of Medicine, Division of Pediatric Hematology, Ankara, Turkey

**Keywords:** vitamin B12, Transcobalamin II, Novel mutation, Novel deletion, Vacuolization

## Abstract

**Objective:**

Transcobalamin II deficiency is one of the rare causes of inherited vitamin B12 disorders in which the patients have characteristically normal or high vitamin B12 levels related to the transport defect of vitamin B12 into the cell, ending up with intracellular cobalamin depletion and high homocysteine and methylmalonic acid levels.

**Materials and Methods:**

Herein, we describe the findings at presentation of four patients who were diagnosed to have transcobalamin II deficiency with novel mutations.

**Results:**

These patients with transcobalamin II deficiency were found to have novel mutations, of whom 2 had the same large deletion (homozygous c.1106+1516-1222+1231del).

**Conclusion:**

Transcobalamin II deficiency should be considered in differential diagnosis of any infant with pancytopenia, failure to thrive, diarrhea, and vomiting.

## Abstract (tr)

**Amaç:**

Transkobalamin II eksikliği nadir bir kalıtsal B12 vitamini bozukluğudur. Defektin B12 vitamininin transportu ile ilgili olması nedeniyle hastalar normal ya da yüksek B12 vitamini düzeylerine eşlik eden yüksek homosistein ve metilmalonik asit düzeylerine sahiptir.

**Gereç ve Yöntemler:**

Bu çalışmada transkobalamin II eksikliği tanısı alan dört hasta sunulmuştur. Bu hastalarda daha önce bildirilmemiş yeni mutasyonlar saptanmıştır.

**Bulgular:**

Hastaların ikisinde aynı büyük delesyon olduğu görülmüştür (homozigot c.1106+1516-1222+1231del).

**Sonuç:**

Pansitopeni, büyüme geriliği, ishal ya da kusması olan tüm bebeklerde transcobalamin II eksikliği ayırıcı tanıda düşünülmelidir.

## INTRODUCTION

Among the pancytopenia etiologies during infancy, the acquired vitamin B12 deficiency in exclusively breast-fed infants of strictly vegan mothers and inherited vitamin B12 deficiency related to transcobalamin II deficiency should be considered, since the treatment of both conditions is easy and possibly life-saving [1,2]. About 30% of plasma cobalamin is bound to transcobalamin II while the remaining part is bound to haptocorrin, but only the part of circulating cobalamin attached to transcobalamin II is the biologically active form and transcobalamin II mediates the entry of cobalamin into a variety of cell types other than hepatocytes [3,4,5]. Transcobalamin II deficiency is a rare autosomal recessive disorder causing intracellular cobalamin depletion, which in turn causes megaloblastic bone marrow failure, accumulation of homocysteine and methylmalonic acid with clinical findings of failure to thrive, diarrhea, vomiting, pancytopenia, megaloblastic anemia, and neurological findings [2]. Homozygous or compound heterozygous mutations in the transcobalamin II gene on chromosome 22q12.2 that contains 9 coding exons are known to cause transcobalamin II deficiency, including deletions, nonsense mutations, and a mutation resulting in activation of a cryptic intronic splice site [6,7,8,9,10,11,12].

Herein, we describe the clinical findings at presentation and outcome of 4 patients with genetically confirmed novel transcobalamin II gene mutations, of whom 3 had large deletions of 1 kb and 1 had a homozygous Q36X mutation.

## MATERIALS AND METHODS

The clinical and laboratory findings of the patients at presentation are summarized in Table 1. The patients were further investigated for molecular diagnosis.

## RESULTS

### Case 1

A 2-month-old girl from the southeastern part of Turkey presented with failure to thrive (birth weight unknown; 2-month-old weight in 10th percentile, length in 25th percentile, head circumference in 3rd to 10th percentiles), irritability, and diarrhea for the last 20 days and was found to have pallor, petechial rash, and no head control upon physical examination. She was the 6th child of first-degree cousins from the 8th gestation, and family history revealed that a sister of hers had died at 1 year of age with diarrhea and vomiting and a brother had died at 3.5 months with bleeding. Liver and renal function tests were unrevealing. Urinalysis revealed absence of proteinuria. Bone marrow aspiration indicated megaloblastic changes in the erythroid and myeloid lineages and vacuolization in the myeloid lineage. Serum vitamin B12 level was found to be 351 pg/mL (normal range: 200-860); however, serum homocysteine was 40 µmol/L (normal: 5.5-17) and urinary methylmalonic acid level was twice the normal value. She was given erythrocyte and platelet transfusions on the first day of admission and intramuscular hydroxocobalamin was initiated at 1000 µg/day with a possible diagnosis of transcobalamin II deficiency. The hemogram findings on the day of vitamin B12 treatment initiation were as follows; RBC: 2.6x1012/L, Hb: 7.4 g/dL, Hct: 21.3%, MCV: 80 fL, WBC: 3.8x109/L, platelets: 61x109/L, absolute neutrophil count (ANC): 0.3x109/L, and absolute lymphocyte count (ALC): 3.4x109/L. By the 6th day of admission the diarrhea subsided and on the 10th day of admission the hemogram results improved to Hb: 8.9 g/dL, Hct: 24.4%, MCV: 78.5 Fl, WBC: 33.2x109/L, platelets: 125x109/L, and ANC: 22.3x109/L. Leukocytosis developed in the absence of an infection after the initiation of vitamin B12 treatment and subsided to the normal range in 2 weeks. Hydroxocobalamin dosage was continued intramuscularly on alternating days for the 2nd week and weekly after the 3rd week. Folic acid at 1 mg orally was added to the treatment. Molecular analyses revealed c.1106+1516-1222+1231del in a homozygous state, which was a deletion of 5304 bp beginning 1516 bp into intron 7 and ending 1231 bp into intron 8, causing deletion of all of exon 8 and a frameshift to produce a premature stop 4 codons into the new reading frame. During the follow-up, the family was learned to have attempted to cease the treatment by their own intention and the patient had similar attacks of pancytopenia and diarrhea. Both attacks resolved after reinitiation of hydroxocobalamin. The patient was also detected to have β-thalassemia trait (HbA2 6%) during outpatient visits due to MCV values as low as 65.2 fL after initiation of vitamin B12 in the absence of iron deficiency. She is currently alive and asymptomatic at 4 years of age.

### Case 2

A 28-day-old boy from the 1st gestation of a couple of first-degree cousins presented with failure to thrive, poor feeding, and vomiting. He was from the Central Anatolia region of Turkey. Hemogram results revealed pancytopenia. Antibiotic treatment was started empirically. He received transfusions several times, and the bone marrow examination was remarkable for megaloblastic changes and vacuolization in bone marrow precursors. Serum vitamin B12 was 623 pg/mL (normal: 200-860). Cyanocobalamin (1000 µg) was initiated intramuscularly with a possible diagnosis of transcobalamin II deficiency. Signs and symptoms declined after cyanocobalamin initiation. Folic acid was added to the vitamin B12 treatment. The control for bone marrow aspiration after vitamin B12 initiation revealed the disappearance of megaloblastic changes and vacuolization in the myeloid lineage. The molecular analyses was ordered and revealed c.1107-347_1222+981del in 364. This complex mutation appears to be a 1444-bp deletion that includes exon 8. There was also a 364-bp insertion. He is currently alive at 6.5 years of age under weekly intramuscular cyanocobalamin.

### Case 3

A 2-month-old girl, from the 1st gestation of a couple of first-degree cousins, presented with diarrhea, vomiting, and fever for 1 week. Body weight and length were below the 3rd percentile for age. Hemogram results revealed pancytopenia. Bone marrow examination revealed megaloblastic changes. Sweat test by pilocarpine iontophoresis was ordered for the diarrhea and results were positive, with a sweat chloride of 87 mEq/L. Molecular testing for cystic fibrosis for the common 21 mutations in Turkey was negative. Serum vitamin B12 was within normal laboratory limits, whereas urinary methylmalonic acid level was 16.45 mmol/mol creatinine (normal: 0-10). Molecular study revealed homozygous c.106C>T p.Q36X. A C-to-T substitution at nucleotide 106 resulted in a premature stop codon. She was started on intramuscular cyanocobalamin at 1000 µg/day on the 13th day of admission; by the 16th day of admission, she was discharged after resolution of symptoms with hemogram findings of Hb: 10.1 g/dL, WBC: 20x109/L, MCV: 89 fl and platelets: 850x109/L, with continuation of treatment twice weekly. Oral folic acid was also initiated. The sweat test was repeated during that period and was normal. She is currently alive at 5 years of age and asymptomatic under weekly cyanocobalamin treatment.

### Case 4

A 3-month-old boy of Turkish origin from Cyprus presented with failure to thrive and poor feeding. Blood and bone marrow examination revealed pancytopenia, hypersegmentation, and megaloblastic changes in the myeloid lineage. Serum homocysteine and vitamin B12 levels were 46 µmol/L (normal: 5.5-17) and 677 pg/mL (normal: 200-860), respectively. Cyanocobalamin was initiated intramuscularly and the pancytopenia resolved. Molecular analyses revealed c.1106+1516-1222+1231del in a homozygous state. The mutation was the same as that found in Case 1.

## DISCUSSION

Transcobalamin II deficiency is a severe disorder with intracellular cobalamin depletion [2]. Transcobalamin II deficiency usually presents with hematological features that overlap with vitamin B12 deficiency including pancytopenia and megaloblastic anemia with high serum homocysteine and methylmalonic acid levels; however, serum vitamin B12 levels are typically normal [13,14,15]. The early initiation of treatment is very important, since pancytopenia and gastrointestinal symptoms including vomiting and diarrhea reverse very soon after treatment, and delay in diagnosis and treatment may cause morbidities and mortalities related to pancytopenia including bleeding and infection in addition to severe and possibly permanent neurological and retinal impairment [14]. Treatment is suggested as hydroxocobalamin or cyanocobalamin either orally and twice weekly or systemically and weekly with high doses of 1000 µg in order to achieve serum cobalamin levels of 1000-10.000 pg/mL, so that cobalamin can be transferred into the cell in the absence of transcobalamin in such high serum levels [15]. Folic acid may be added to the treatment [15].

In cases 1 and 2, the bone marrow findings of vacuolization in the myeloid lineage is interesting. Vacuolization is an important finding in another metabolic disease, namely Pearson syndrome, that may present with pancytopenia, megaloblastic anemia during infancy with lactic acidosis, and exocrine pancreas dysfunction related to a mitochondrial defect [16]. In the literature, Ratschmann et al. provided the bone marrow figures of their index patient of 6 weeks old with transcobalamin II deficiency and described the changes in the myeloid lineage as dysgranulopoiesis [12]. In those findings, vacuolization was prominent, similar to our patients (cases 1 and 2). Vacuolization may be an additional finding of transcobalamin II deficient patients that may be related to defect in the mitochondrial DNA synthesis, as well, resulting from cobalamin deficiency.

Case 1 of the current report had an initial MCV value of 88 fL; after vitamin B12 treatment, the patient had MCV measured as low as 67.2 fL and was further tested with hemoglobin electrophoresis. She was found to have β-thalassemia trait. This indicates that initial MCV values may not be macrocytic in the presence of β-thalassemia trait; if the clinical presentation is very suggestive of transcobalamin II deficiency, the normal MCV values may not preclude the diagnosis. Additionally, since case 2 was presented at the neonatal stage, MCV was already macrocytic. These findings may indicate that a normal MCV for age may not exclude macrocytic anemia etiologies.

Another finding is that in cases 1 and 3, after the initiation of vitamin B12, hematological improvement occurred with rapid and dramatic leukocytosis in case 1 and leukocytosis and thrombocytosis in case 3. In both cases the high counts normalized in follow-up, but our patients indicate that initiation of therapy may cause a rapid increase of blood counts in transcobalamin II deficient patients.

Additionally, case 3 had a transiently high sweat chloride level that normalized after vitamin B12 treatment. Among the etiologies that may cause a false-positive sweat chloride test, transcobalamin II deficiency has not been reported [17,18]. Transcobalamin II deficiency may be one of the causes of false-positive sweat tests that has not been previously reported and this hypothesis may require further support from additional studies.

In cases 1, 2, and 4, patients were found to have large deletions, and in case 3 a point mutation was detected, all of which are reported here as novel findings. Tanner et al. previously reported the same mutation among their juvenile cobalamin deficiency patients with GIF mutations together with Yassin et al. [19,20]. Both of those patients with GIF mutations were of African ancestry, and Tanner et al. claimed that the mutation might be common in some African populations through a founder effect [19]. The same hypothesis may also be true for our patients (cases 1 and 4) who have the same novel mutation, indicating a common mutation among the Turkish population.

In conclusion, vitamin B12 deficiency has deleterious long-term consequences and, differing from nutritional deficiencies of vitamin B12, patients with transcobalamin II deficiency are especially responsive to high doses of vitamin B12 [21]. Transcobalamin II deficiency should be considered in differential diagnosis of any infant with pancytopenia, failure to thrive, diarrhea, and vomiting. In patients with pancytopenia, transcobalamin II deficiency should be considered in differential diagnosis, especially in countries with high rates of consanguineous marriages, like Turkey. Early initiation of high-dose vitamin B12 treatment is very crucial not only for being potentially life-saving, but also in order to prevent long-term neurological morbidities.

## Figures and Tables

**Table 1:**
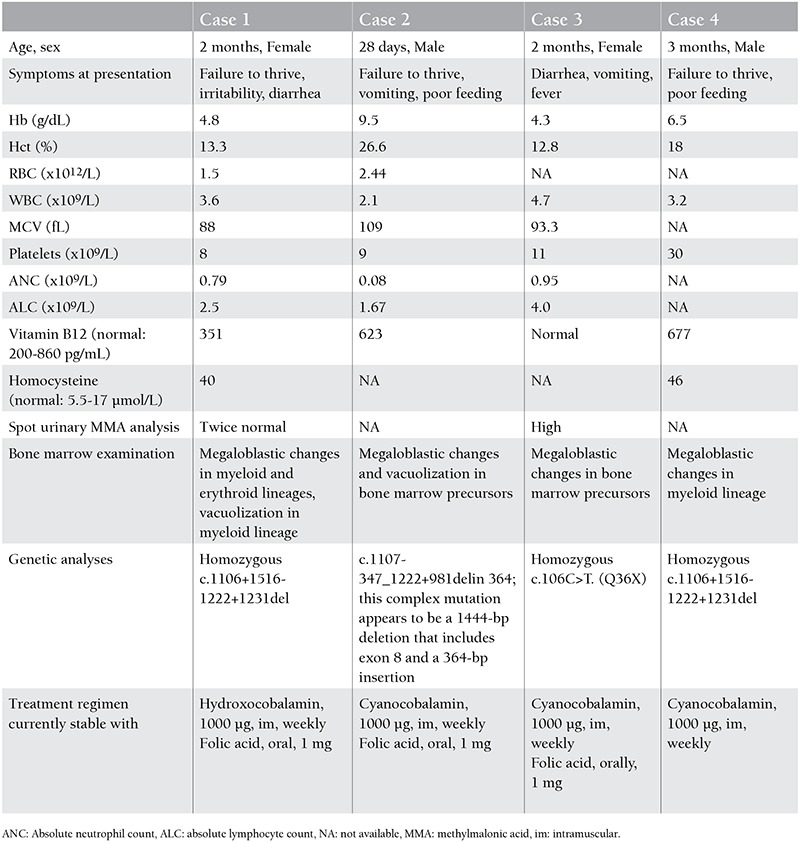
Clinical and laboratory findings of patients at presentation.

## References

[1] Kanra G, Cetin M, Unal S, Haliloglu G, Akça T, Akalan N, Kara A (2005). Answer to hypotonia: a simple hemogram. J Child Neurol.

[2] Schiff M, Bard G, Barlogis V, Hamel C, Moat SJ, Odent S, Shortland G, Touati G, Giraudier S (2010). Should transcobalamin deficiency be treated aggressively?. J Inherit Metab Dis.

[3] Rosenblatt DS, Fenton WA, In: Budet AL, Valle D, Sly W (eds) (2001). Inherited disorders of folate and cobalamin transport and metabolism. The Metabolic and Molecular Bases of Inherited Disease.

[4] Oberley MJ, Yang DT (2013). Laboratory testing for cobalamin deficiency in megaloblastic anemia. Am J Hematol.

[5] Meyers PA, Carmel R (1984). Hereditary transcobalamin II deficiency with subnormal serum cobalamin levels. Pediatrics.

[6] Arwert F, Porck HJ, Frater-Schröder M, Brahe C, Westerveld A, Meera Khan P, Zang K, Frants RR, Kortbeek HE, Erikkson AW (1986). Assignment of human transcobalamin II (TC2) to chromosome 22 using somatic cell hybrids and monosomic meningioma cells. Hum Genet.

[7] Regec A, Quadros EV, Platica O, Rothenberg SP (1995). The cloning and characterization of the human transcobalamin II gene. Blood.

[8] Li N, Seetharam S, Seetharam B (1995). Genomic structure of human transcobalamin II: comparison to human intrinsic factor and transcobalamin I. Biochem Biophys Res Commun.

[9] Li N, Rosenblatt DS, Kamen BA, Seetharam S, Seetharam B (1994). Identification of two mutant alleles of transcobalamin II in an affected family. Hum Mol Genet.

[10] Li N, Rosenblatt DS, Seetharam B (1994). Nonsense mutations in human transcobalamin II deficiency. Biochem Biophys Res Commun.

[11] Namour F, Helfer AC, Quadros EV, Alberto JM, Bibi HM, Orning L, Rosenblatt DS, Jean-Louis G (2003). Transcobalamin deficiency due to activation of an intra exonic cryptic splice site. Br J Haematol.

[12] Ratschmann R, Minkov M, Kis A, Hung C, Rupar T, Mühl A, Fowler B, Nexo E, Bodamer OA (2009). Transcobalamin II deficiency at birth. Mol Genet Metab.

[13] Hakami N, Neiman PE, Canellos GP, Lazerson J (1971). Neonatal megaloblastic anemia due to inherited transcobalamin II deficiency in two siblings. N Engl J Med.

[14] Hall CA (1992). The neurologic aspects of transcobalamin II deficiency. Br J Haematol.

[15] Watkins D, Whitehead VM, Rosenblatt D, In: Nathan DG, Orkin SH (eds) (2009). Megaloblastic anemia. Nathan and Oski’s Hematology of Infancy and Childhood.

[16] Tumino M, Meli C, Farruggia P, La Spina M, Faraci M, Castana C, Raimondo V, Alfano M, Pittalà A, Lo Nigro L, Russo G, Cataldo A (2011). Clinical manifestations and management of four children with Pearson syndrome. Am J Med Genet A.

[17] Mishra A, Greaves R, Massie J (2005). The relevance of sweat testing for the diagnosis of cystic fibrosis in the genomic era. Clin Biochem Rev.

[18] Boat TF, Acton JD, In: Kliegman RM, Behrman RE, Jenson HB, Stanton BF (eds) (2007). Cystic fibrosis. Nelson Textbook of Pediatrics.

[19] Tanner SM, Li Z, Perko JD, Oner C, Cetin M, Altay C, Yurtsever Z, David KL, Faivre L, Ismail EA, Gräsbeck R (2005). Hereditary juvenile cobalamin deficiency caused by mutations in the intrinsic factor gene. Proc Natl Acad Sci USA.

[20] Yassin F, Rothenberg SP, Rao S, Gordon MM, Alpers DH, Quadros EV (2004). Identification of a 4-base deletion in the gene in inherited intrinsic factor deficiency. Blood.

[21] Evim MS, Erdöl Ş, Özdemir Ö, Baytan B, Güneş AM (2011). Long-term outcome in children with nutritional vitamin B12 deficiency. Turk J Hematol.

